# Supplementary material for: Measuring quality of family planning counselling and its effects on uptake of contraceptives in public health facilities in Uttar Pradesh, India: A cross-sectional analysis
Source: PLoS One. 2021 May 4;16(5):e0239565. doi: 10.1371/journal.pone.0239565 (PMC8096066; doi:10.1371/journal.pone.0239565)
Supplement: S1 Appendix — (DOCX) [file pone.0239565.s001.docx]

**S1 Appendix. Rotated Components Matrix.**

| **Individual Items to assess Quality of Counselling on Family Planning** | **Comp 1** | **Comp 2** | **Comp 3** | **Comp 4** |
| --- | --- | --- | --- | --- |
| Provider asked women about their fertility goals | 0.31 |  |  |  |
| Provider asked women about any problems women faced while using FP methods earlier | 0.38 |  |  |  |
| Provider asked women about their preferred method | 0.40 |  |  |  |
| Provider asked women about different methods they had used earlier | 0.36 |  |  |  |
| Provider told women about different FP methods | 0.37 |  |  |  |
| Provider explained women how to use the method selected by them | 0.40 |  |  |  |
| Provider explained the side-effects of the selected methods to women |  |  | -0.46 |  |
| Provider counselled women on what to do in case they face any problems in using the selected method | 0.41 |  |  |  |
| Provider encouraged women to ask questions |  |  | 0.60 |  |
| Women reported that providers spent sufficient time in counselling |  |  | 0.59 |  |
| Women reported being treated in a friendly manner by providers |  | 0.66 |  |  |
| Women reported being treated respectfully by providers |  | 0.70 |  |  |
| Providers did not apply any pressure on women to select a particular method |  |  |  | 0.94 |
